# Supplementary material for: Defining the cell and molecular origins of the primate ovarian reserve
Source: Nat Commun. 2025 Aug 26;16:7539. doi: 10.1038/s41467-025-62702-0 (PMC12381125; doi:10.1038/s41467-025-62702-0)
Supplement: Supplementary file 2 — Description of Additional Supplementary Files [file 41467_2025_62702_MOESM2_ESM.pdf]

Description of Additional supplementary files

Supplementary Data 1. Microsoft Excel file associated with Figure 2 and Supplementary Figure 3.

(A) Differential gene expression (DEG) analysis tables from Visium CytAssist analysis comparing W5 ovaries (W5_3, W5_4) to mesonephros and adrenal regions and to all tissues on the section. Threshold: pval_adj 1.

(B) CytAssist DEG comparisons of W6 ovaries (W6_2, W6_3) to mesonephros and adrenal regions and to all tissues on the section. Threshold: pval_adj 1.

(C) CytAssist DEG comparisons of W5 ovaries to each other and to W6 ovaries. Threshold: pval_adj 1.

(D) CytAssist DEG comparisons of W5 and W6 ovaries to W5 testes (W5_1, W5_2) and W6 testis (W6_1). Threshold: pval_adj 1. E: Cluster analysis of NanoString CosMx cell type clusters from W6 sections. Threshold: pval_adj <0.05.

Supplementary Data 2. Microsoft Excel file associated with Figure 3 and Supplementary Figure 5. (A) DEG comparisons following pseudobulk analysis of W8 stroma, granulosa and undefined cell clusters. Threshold: FDR

Supplementary Data 3. Microsoft Excel file associated with Figure 3 and Supplementary Figure 7. A: Genes in granulosa clusters from UMAP plot in Figure. 3E. Threshold: pval_adj <0.05.
