# Supplementary material for: Defining the cell and molecular origins of the primate ovarian reserve
Source: Nat Commun. 2025 Aug 26;16:7539. doi: 10.1038/s41467-025-62702-0 (PMC12381125; doi:10.1038/s41467-025-62702-0)
Supplement: Supplementary file 6 — Reporting summary [file 41467_2025_62702_MOESM6_ESM.pdf]

nature portfolio

Corresponding author(s): Amander T. Clark

Last updated by author(s): Jul 9, 2025

Reporting Summary

Nature Portfolio wishes to improve the reproducibility of the work that we publish. This form provides structure for consistency and transparency in reporting. For further information on Nature Portfolio policies, see our Editorial Policies and the Editorial Policy Checklist.

Statistics

For all statistical analyses, confirm that the following items are present in the figure legend, table legend, main text, or Methods section.

n/a	Confirmed	
☐	☒	The exact sample size (*n*) for each experimental group/condition, given as a discrete number and unit of measurement
☐	☒	A statement on whether measurements were taken from distinct samples or whether the same sample was measured repeatedly
☐	☒	The statistical test(s) used AND whether they are one- or two-sided<br>*Only common tests should be described solely by name; describe more complex techniques in the Methods section.*
☐	☒	A description of all covariates tested
☐	☒	A description of any assumptions or corrections, such as tests of normality and adjustment for multiple comparisons
☐	☒	A full description of the statistical parameters including central tendency (e.g. means) or other basic estimates (e.g. regression coefficient) AND variation (e.g. standard deviation) or associated estimates of uncertainty (e.g. confidence intervals)
☐	☒	For null hypothesis testing, the test statistic (e.g. *F*, *t*, *r*) with confidence intervals, effect sizes, degrees of freedom and *P* value noted<br>*Give P values as exact values whenever suitable.*
☒	☐	For Bayesian analysis, information on the choice of priors and Markov chain Monte Carlo settings
☒	☐	For hierarchical and complex designs, identification of the appropriate level for tests and full reporting of outcomes
☒	☐	Estimates of effect sizes (e.g. Cohen's *d*, Pearson's *r*), indicating how they were calculated

Our web collection on statistics for biologists contains articles on many of the points above.

Software and code

Policy information about availability of computer code

Data collection	No code was used for data collection.
Data analysis	Data was analysed in the R software environment (version 4.2.2) using the Seurat package (v4 or v5 as indicated) standard pipelines with default parameters; a summary of the analysis is included in the Methods section. Code used for sample processing and analysis is provided in the following Github repository (https://github.com/ejscience/2025_Wamaitha_Rhesus_OvarianReserve).

For manuscripts utilizing custom algorithms or software that are central to the research but not yet described in published literature, software must be made available to editors and reviewers. We strongly encourage code deposition in a community repository (e.g. GitHub). See the Nature Portfolio guidelines for submitting code & software for further information.

Data

Policy information about availability of data

All manuscripts must include a data availability statement. This statement should provide the following information, where applicable:

- Accession codes, unique identifiers, or web links for publicly available datasets
- A description of any restrictions on data availability
- For clinical datasets or third party data, please ensure that the statement adheres to our policy

The single cell RNA sequencing datasets generated for the current study are available in the Gene Expression Omnibus (GEO) under accession number GSE263989 [https://www.ncbi.xyz/geo/query/acc.cgi?acc=GSE263989]. The spatial transcriptomics datasets are available on Zenodo [https://zenodo.org/records/15477421].

Published datasets for cynomolgus macaque samples were downloaded from GEO including GSE16004318 [https://www.ncbi.nlm.nih.gov/geo/query/acc.cgi?acc=GSE160043], GSE19426461 [https://www.ncbi.nlm.nih.gov/geo/query/acc.cgi?acc=GSE194264] and GSE14962920 [https://www.ncbi.nlm.nih.gov/geo/query/acc.cgi?acc=GSE149629] or Zenodo 691835516 [https://zenodo.org/records/6918355].

Research involving human participants, their data, or biological material

Policy information about studies with human participants or human data. See also policy information about sex, gender (identity/presentation), and sexual orientation and race, ethnicity and racism.

Reporting on sex and gender	n/a
Reporting on race, ethnicity, or other socially relevant groupings	n/a
Population characteristics	n/a
Recruitment	n/a
Ethics oversight	n/a

Note that full information on the approval of the study protocol must also be provided in the manuscript.

Field-specific reporting

Please select the one below that is the best fit for your research. If you are not sure, read the appropriate sections before making your selection.

☒ Life sciences ☐ Behavioural & social sciences ☐ Ecological, evolutionary & environmental sciences

For a reference copy of the document with all sections, see nature.com/documents/nr-reporting-summary-flat.pdf

Life sciences study design

All studies must disclose on these points even when the disclosure is negative.

Sample size	No sample size calculation was performed. We collected and analysed 1 - 3 biological replicates per gestation timepoint; the number used for each figure or analysis is noted in the legend or Methods. Cell counting using immunofluorescence images was performed at least in triplicate (n=3) as this is sufficient for statistical analysis.
Data exclusions	ONPRC017_A single cell RNA-seq library was excluded from downstream analysis as did not pass quality control metrics (see Methods).
Replication	Biological replicates were collected at each timepoint; 2 technical replicates were collected for single cell RNA seq library prep.
Randomization	n/a
Blinding	n/a

Reporting for specific materials, systems and methods

We require information from authors about some types of materials, experimental systems and methods used in many studies. Here, indicate whether each material, system or method listed is relevant to your study. If you are not sure if a list item applies to your research, read the appropriate section before selecting a response.

Materials & experimental systems

n/a	Involved in the study	
☐	☒	Antibodies
☒	☐	Eukaryotic cell lines
☒	☐	Palaeontology and archaeology
☐	☒	Animals and other organisms
☒	☐	Clinical data
☒	☐	Dual use research of concern
☒	☐	Plants

Methods

n/a	Involved in the study	
☒	☐	ChIP-seq
☒	☐	Flow cytometry
☒	☐	MRI-based neuroimaging

Antibodies

Antibodies used	Antibodies used in the study are listed in Extended Data Table 3.
Validation	Antibodies were indicated as validated for use in rhesus macaque on manufacturers website, were previously published, or were tested for the first time in this paper - validation was determined as positive specific staining of the target cell type with low/no background.

Animals and other research organisms

Policy information about studies involving animals; ARRIVE guidelines recommended for reporting animal research, and Sex and Gender in Research

Laboratory animals	Rhesus macaques (Macaca mulatta); 14 breeding age females were paired with males resulting in 16 offspring, collected via C-section (2 dams had two separate pregnancies). See Methods for details.
Wild animals	n/a
Reporting on sex	Primarily XX tissues were collected for this study due to the focus on ovarian gonadal development (n=2 at CS16, CS20; n = 3 at CS23, D100 and D130). XY tissues were collected at early timepoints for comparative analysis (n=2 at CS16, n =1 at CS20) . Foetal sex was determined using PCR on either tissue collected at necropsy or circulating cell-free DNA isolated from maternal blood; see Methods for details.
Field-collected samples	n/a
Ethics oversight	Rhesus macaque time-mated breeding experiments were conducted following the approval of the ONPRC Institutional Animal Care and Use Committee (IACUC) with secondary approval by the UCLA Chancellors Animal Research Committee for all experiments with rhesus tissues performed at UCLA.

Note that full information on the approval of the study protocol must also be provided in the manuscript.

Plants

Seed stocks	n/a
Novel plant genotypes	n/a
Authentication	n/a
